# Implication of Netrin-1 Gain of Expression in Canine Nodal Lymphoma

**DOI:** 10.3390/vetsci9090494

**Published:** 2022-09-10

**Authors:** Antonin Tortereau, Nadège Milhau, Elodie Rhumy, Marie Castets, Frédérique Ponce, Patrick Mehlen, Thierry Marchal

**Affiliations:** 1 Université de Lyon, VetAgro Sup, Interactions Cellules Environnement, ICE, 69280 Marcy l’Etoile, France; 2 Childhood Cancer & Cell Death (C3), Université Claude Bernard Lyon 1, INSERM 1052, CNRS 5286, Centre Léon Bérard, Centre de Recherche en Cancérologie de Lyon (CRCL), 69008 Lyon, France; 3 Université de Lyon, VetAgro Sup, Service de Cancérologie, 69280 Marcy l’Etoile, France; 4 Apoptosis, Cancer and Development Laboratory—Equipe labellisée ‘La Ligue’, LabEx DEVweCAN, Centre de Recherche en Cancérologie de Lyon, INSERM U1052-CNRS UMR5286, Université de Lyon 1, Centre Léon Bérard, 69008 Lyon, France; 5 Department of Research and Innovation, Centre Léon Bérard, 69008 Lyon, France

**Keywords:** nodal lymphoma, dog, netrin-1, dependence receptors, immunotherapy

## Abstract

**Simple Summary:**

Canine lymphomas represent one of the most frequent groups of neoplasia, for which prognosis may be poor. Treatments are based on polychemotherapy, with variable responses. As in human lymphomas, more and more targeted therapies are studied and developed. Therapy to restore apoptosis in neoplastic cells is one of them. Netrin-1 is a ligand of dependence receptors. When bound to its receptor, a positive signaling is triggered. When unbound, apoptosis is induced. In some human cancers, neoplastic cells can lose the ability to induce apoptosis by overexpressing netrin-1, or by decreasing the receptor expression. We hypothesized a similar pathway in canine lymphomas. We observed increased expression of netrin-1, particularly in high-grade nodal lymphomas. In vitro evaluation of an anti-netrin-1 antibody is encouraging as apoptosis is restored in a T-cell lymphoma cell line. Netrin-1 appears thus as a possible survival factor in dog lymphomas. This study suggests it can be a promising tool for a targeted therapy in lymphoma management in dogs.

**Abstract:**

Netrin-1 is a member of the laminin superfamily, and is known to interact with specific receptors, called dependence receptors. While upon netrin-1 binding these receptors initiate positive signaling, in absence of netrin-1, these receptors trigger apoptosis. Tumor cells can avoid apoptosis by inactivating these receptors or by gaining ligand expression. The aim of the present study was to investigate the expression of netrin-1, the ligand of dependence receptors, in canine healthy lymph nodes (LN), and in lymphomas and to evaluate efficiency of a netrin-1 interfering compound in cell cultures from canine lymphoma. Thirty-two control LN and 169 lymphomas were analyzed through immunohistochemistry. Netrin-1 was expressed in the nucleoli of lymphoid and non-lymphoid cells in controls. Acquisition of a cytoplasmic expression was present in B-cell lymphomas (23.1 % in low-grade and 50.6% in high-grade) and T-cell lymphomas (50.0 % in low-grade and 78.8 % in high-grade), with a significant difference between the high- and low-grade in B-cell lymphomas. Through flow cytometry, we showed a significant increase in netrin-1 expression in either high-grade B-cell and T-cell lymphomas (19 and 5, respectively) compared with healthy LN (5), likewise an RT-qPCR analysis demonstrated a significant increase in netrin-1 expression level in 14 samples of lymphomas compared with eight samples of healthy LN. A T-cell aggressive canine lymphoma cell line and four primary canine nodal lymphomas cell cultures were treated with a netrin-1 interfering antibody. Apoptosis by measuring caspase 3 activity was significantly increased in the cell line and viability was decreased in three of the four primary cell cultures. Together, these data suggest that netrin-1 expression is increased in lymphoma, and more specifically in high-grade lymphomas, and that netrin-1 can act as a survival factor for the neoplastic cells, and so be a therapeutic target.

## 1. Introduction

Netrins gather a family of proteins first identified in the nematode worm *Caenorhabditis elegans* [1,2,3,4]. To date, 3 secreted netrins, netrin-1, -3 and -4, and 2 glycosylphosphatidylinositol (GPI)-anchored membrane proteins, netrin-G1 and -G2, have been identified in mammals [1,2,5]. All netrins are members of the laminin superfamily.

During embryonic development, netrin-1 is expressed by floor plate cells in the ventral side of the embryonic spinal cord and is responsible for the attraction of commissural neurons towards the floor plate. The netrin-1 receptor Deleted in Colorectal Cancer (DCC) is required for this attraction. Netrin-1 also acts as a regulator of neuron migration, notably during cerebellar development [1]. Finally, a role for netrin-1 outside the developing nervous system has also been shown, notably during mammary gland development or in angiogenesis [6,7,8].

Membrane receptors were classically considered to be active only in the presence of their ligand, and to remain inactive in their absence. However, some receptors have been shown to induce two signaling pathways: whereas ligand binding triggers a positive signaling leading to cell proliferation, migration or differentiation, these receptors are not inactive in the absence of their ligand but rather induce cell death through apoptosis. These receptors are named “dependence receptors” [4,9,10]. Netrin-1 receptors DCC and UNCoordinated 5 homologs (UNC5A-D) belong to the dependence receptors family [1,2,5,11]. Indeed, in absence of netrin-1, DCC and UNC5 receptors actively induce apoptosis [9,10,11,12].

The dependence receptors functioning model allows to postulate that they can play a role in tumor prevention, due to their ability to promote apoptosis when unbound to their ligand. Indeed, it has been shown that dependence receptors act as tumor suppressor genes, especially for netrin-1 dependence receptors DCC and UNC5C [13,14,15].

Tumor cells can bypass this dependence pathway, either by inactivating dependence receptor expression itself or through acquisition of ligand expression. This gain of netrin activity is proven to be a selective advantage for tumor cells survival, both in vitro and in vivo, by using compounds inhibiting/interfering with netrin-1/receptors interactions, especially in some human lymphomas [16]. Therefore, alternative therapies can be promising tools in lymphoma management.

Non-Hodgkin lymphomas are common malignancies in dogs, with an estimated incidence of 33 per 100,000, without difference in age or sex distribution [17,18]. As in humans, lymphomas mainly involve lymph nodes (>80 %), either locally or generally. Extranodal sites include skin (>12%), and less commonly the digestive tract, spleen, tonsils, and eyes. Clinical signs are similar between humans and dogs and it is suggested that humans and dogs share common pathways or an ancestrally retained pathogenesis basis for lymphoma development [19,20].

In dogs, the therapeutic approach is mainly based on conventional polychemotherapy, with variable responses [21]. As in humans, targeting the dependence receptors pathway can prove to be interesting in this tumor management, alongside other targeted therapies [22].

Thus, the aims of this project were twofold: first, to determine the involvement of a gain of netrin-1 expression in canine lymphomas in lymph nodes by analyzing netrin-1 expression levels in canine lymphoma samples; then, to establish in vitro efficiency of netrin-1 interfering compounds in canine lymphomas treatment.

## 2. Materials and Methods

### 2.1. Case Selection

Thirty-two normal lymph nodes were collected at VetAgro Sup during necropsies or surgeries. For eight of them, one half was frozen during 1 min in isopentan cooled by liquid nitrogen and stored at −80 °C until RT-qPCR analysis and the second half was fixed in 10% neutral buffered formalin and paraffin-embedded tissues (for immunohistochemistry studies). For the 24 remaining normal lymph nodes, only fixation in 10% neutral buffered formalin and paraffin embedding was achieved for immunohistochemistry studies. Histological assessment of the absence of any visible lesions was made on a Hematoxylin–Eosin (H.E.)-stained slide before selection in the study.

One hundred and sixty-nine canine nodal lymphomas were collected at VetAgro Sup between 2001 and 2017 for the immunohistochemistry study. For all cases following information were recorded: age, sex, and breed and when available, case history, clinical and paraclinical data, therapy if any, and evolution of the disease. A board-certified pathologist (AT) examined each case using an H.E.-stained slide associated with CD3, CD20, and Ki-67 immunostaining to confirm the diagnosis and type of lymphoma according to Fournel et al. classification criteria [19,23]. Nodal lymphomas were subsequently categorized within four groups: low-grade B-cell lymphoma, high-grade B-cell lymphoma, low-grade T-cell lymphoma, and high-grade T-cell lymphoma. One hundred and nine were B-cell lymphomas (26 low-grade and 83 high-grade) and 60 were T-cell lymphomas (14 low-grade and 46 high-grade whose 13 lymphoblastic).

For twenty-six cases of canine nodal lymphomas (19 B-cell lymphoma (all high-grade) and seven T-cell lymphoma (five high-grade and two low-grade), tumor lymph nodes were aspirated during the extension assessment and stored in RPMI medium before flow cytometry analysis. Five normal superficial inguinal lymph nodes resected in the course of extensive mammary tumor surgery and lacking metastasis, were aspirated and used in the same conditions to serve as control.

For fourteen cases of canine nodal lymphomas (eight B-cell lymphoma and six T-cell lymphoma), tumor lymph nodes were aspirated during the extension assessment and stored in a RNA protect medium (RNAprotect Cell Reagent, Qiagen^®®^, Hilden, Germany) at −80 °C until RT-qPCR analysis.

To determine whether netrin-1 acts as a survival factor for canine lymphoma cells, as expected from its role as a ligand of dependence receptors, we used one nitrogen-frozen cell line derived form a spontaneously occurring aggressive T-cell lymphoma (PER-VAS [24]) expressing netrin-1 (Figure S1) and four primary nodal lymphoma cell cultures. These primary cell cultures were obtained by fine needle aspiration of lymph nodes during the extension assessment. Fine-needle aspirates were placed in RPMI medium. Two diffuse large B-cell lymphomas (DLBCL) (polymorphic centroblastic), one transforming marginal zone B-cell lymphoma, and one small clear-cell T-cell lymphoma were obtained.

All samples were made in compliance with VetAgro Sup ethic committee rules.

### 2.2. Bioinformatic Comparison between Human and Canine Netrin-1

Human and dog Netrin-1 gene and protein sequences were compared using the NCBI database. Sequence alignment between these two species were performed with Seaview software [25]. Canine netrin-1 protein sequences were predicted. Among all 604 amino acids composing the protein in the two species, five amino acids were different, establishing a 98.5% homology between the two sequences. Sequence alignment showed the first two different amino acids were located before the laminin domain, the three others within the NTR domain (Figure S2). Such homology justified the use of antibodies directed against human peptide for our study in dog.

### 2.3. Detection of Netrin-1 Expression by Immunohistochemistry

Immunohistochemical staining for netrin-1 (169 nodal lymphomas and 32 normal lymph nodes) was performed in a manual way, with the avidin-biotin-peroxidase complex method, on formalin-fixed, paraffin-embedded tissues. Netrin-1 expression was evaluated using a netrin-1 rat anti-mouse monoclonal antibody (clone, diluted 1:100, R&D Systems, Minneapolis, MN, USA).

Antigen retrieval was carried out at 95 °C for 40 min in a pH9 solution (Dako target retrieval solution, pH 9, Dako, Carpinteria, CA, USA). Amplification of the labeling was performed by the ultraTek HRP (anti-polyvalent) ready-to-use kit (ScyTek Laboratories, Logan, UT, USA) 30 min at 20 °C and revelation with the Vector NovaRED Peroxidase (HRP) Substrate kit (Vector, Burlingame, CA, USA) for 5 min. After a 3 min counterstaining with Hematoxylin, sections were dehydrated and mounted. For each sample, a negative control was realized, by replacing the anti-netrin-1 antibody with the antibody diluent solution (Emerald diluent antibody, Cellmarque, Sigma Aldrich, CA, USA).

A qualitative and semi-quantitative evaluation of netrin-1 expression was performed. The type of immunostained cell and the staining location were recorded. Semi-quantitative grading of positively labelled lymphoma cells was realized, according to Table 1.

### 2.4. Quantification of Intracellular Netrin-1 by Flow Cytometry

Cells in RPMI medium (26 nodal lymphomas and five normal lymph nodes) were formalin-fixed for 15 min, and permeabilized for 10 min with a 0.3% saponin solution (Sigma Aldrich, France). After washing, cells were incubated for 30 min with netrin-1 rat anti-mouse monoclonal antibody (clone, diluted 1:100, R&D Systems, Minneapolis, USA), and 30 min with a secondary polyclonal goat anti-rat/mouse FITC (clone F047902-2, Agilent, Carpinteria, CA). Fluorescence was measured after an ultimate washing, with a cytometer Accuri (Becton Dickinson, France). Results were analyzed with the BD Accuri C6 Software ^®®^ (Becton Dickinson, Le Pont de Claix, France). To discriminate fluorescence intensity between culture cells, evaluation of difference in mean fluorescence intensity (ΔMFI) was performed, as published in Fellman et al. [26], according to this formula: ΔMFI = (MFI Netrin-1–MFI control) / MFI control. Control cells were incubated with the secondary antibody only.

### 2.5. Quantification of Netrin-1 Expression by RT-qPCR

Total mRNAs were extracted from frozen organ samples (8 normal lymph nodes) and fine-needle aspirates (14 nodal lymphomas) using Nucleospin RNAII kit (Macherey-Nagel, Hoerdt, France) and 1 µg was reverse transcribed using the iScript cDNA Synthesis kit (Bio-Rad, Hercules, CA, USA). A real-time quantitative PCR was performed on a LightCycler 2.0 apparatus (Roche Life Science, city name, France) using the LightCycler FastStart DNA Master SYBERGreen I kit (Roche Life Science, Meylan, France). The hprt housekeeper gene was chosen as an internal control for all amplifications [27].

Primers sequences were designed from the known sequences of canine orthologs of genes of interest, according to Ensembl and NCBI databases (Table 2) [28]. BlastN searches were performed to confirm gene specificity of the newly designed sequences and the absence of DNA polymorphisms.

Thermal cycling conditions comprised an initial denaturation step at 95 °C for 10 min and 45 cycles at 95 °C for 15 s and 67 °C (for netrin-1 amplification) or 65 °C (for hprt amplification) for 1 min.

Products of amplification were checked by electrophoresis on a 2% agarose gel, revealed by a GelDoc XR apparatus (Bio-Rad).

Final results were expressed as differences in target gene expression relative to the hprt gene, according to the formula: 2-ΔCp, with ΔCp = Cptarget gene-Cphprt, expressed in arbitrary units [29].

### 2.6. Lymphoma Cell Cultures and Cell Death Assays

To explore whether netrin-1 acts as a survival factor for canine lymphoma cells we first tested the effect of a netrin-1 interfering monoclonal antibody on a canine T-cell line expressing netrin-1 and considering the result obtained on four primary canine nodal lymphoma cells.

For the T-cell canine lymphoma cell line, 1 × 10^5^ viable cells were cultured during 24 h in RPMI 1640 medium without serum and were treated or not with a netrin-1 interfering monoclonal antibody (20 µg/µL (NetrisPharma^®®^, Lyon, France) alone or in combination with FLAG-tagged netrin-1 (0.15 µg/µL) (Adipogen Life Science, San Diego, CA, USA). The extent of cell apoptosis was monitored by measuring caspase-3 activity with the Caspase-3/CPP32 Fluorometric Assay kit (BioVision, Whaltham, MA, USA) and analyzed as described by the manufacturer. This experiment was performed in triplicate.

The four nodal lymphoma aspirates used for this analysis were seeded at 1 × 10^6^ viable cell per well (3 wells per aspirate) in a 12-well plate. Cultures were performed in 1 mL of RPMI 1640 medium supplemented with 5% fetal bovine serum, 2% penicillin and streptomycin and 1% glutamine (Eurobio, Les Ulis, France) at 37 °C. Twenty four hours after start of culturing, 5 µL of netrin-1 interfering monoclonal antibody (10 µg/µL NetrisPharma^®®^, Lyon, France) was added to well 1, 5 µL of antibody dilution medium was added to well 2 as negative control and 20 µL of 1mM Stauropsorin (Sigma-Aldrich^®®^, Saint-Louis, MO, USA) was added to well 3 as positive control. After 24 h of incubation, the extent of cell apoptosis was monitored by the FITC annexin V/ IP cell apoptosis kit (Invitrogen, Whaltham, MA, USA) and analyzed as described by the manufacturer.

### 2.7. Statistical Analyses

Statistical analyses were made with the R Software [30]. Data distribution was not normal according to Shapiro–Wilk tests. Mann–Whitney–Wilcoxon tests were thus performed to compare results between independent sample tests, or to compare simultaneously several means

## 3. Results

### 3.1. Netrin-1 Expression in Normal Lymph Nodes and in Nodal Lymphomas

#### 3.1.1. Netrin-1 Expression in Normal Lymph Nodes

**Figure 1:**
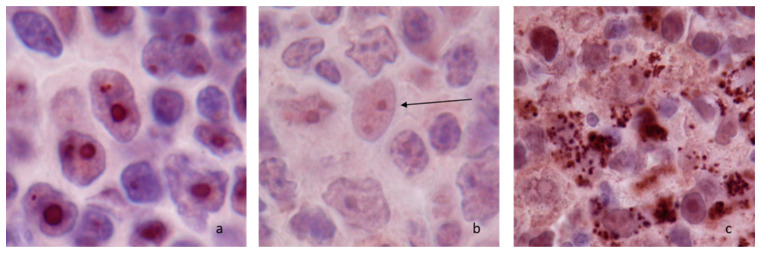
Netrin-1 immunostaining in canine normal lymph nodes (×100) (**a**) nucleolar staining in macronucleolated medium cells of the marginal zone; (**b**) nucleolar staining in follicular dendritic cells (arrow); (**c**) nucleolar and granular cytoplasmic staining in sinus macrophages.

Thirty-two lymph nodes without histological lesions were tested. A diffuse nucleolar immunostaining with peripheral reinforcement was consistently present in both lymphoid and non-lymphoid cell. In lymphoid cells, this nucleolar staining was particularly visible in macronucleolated cells of the marginal zone (Figure 1a). In non-lymphoid cells nucleolar expression included dendritic follicular cells, reticulated cells, macrophages in lymphatic sinuses, and endothelial cells from post-capillary venules (Figure 1b). There was no cytoplasmic netrin-1 expression in lymphoid cells, except in mitotic cells whereas an intense granular cytoplasmic expression was seen in macrophages in medullary sinuses (Figure 1c, Figure S3).

#### 3.1.2. Netrin-1 Expression in Nodal Lymphomas

**Figure 2:**
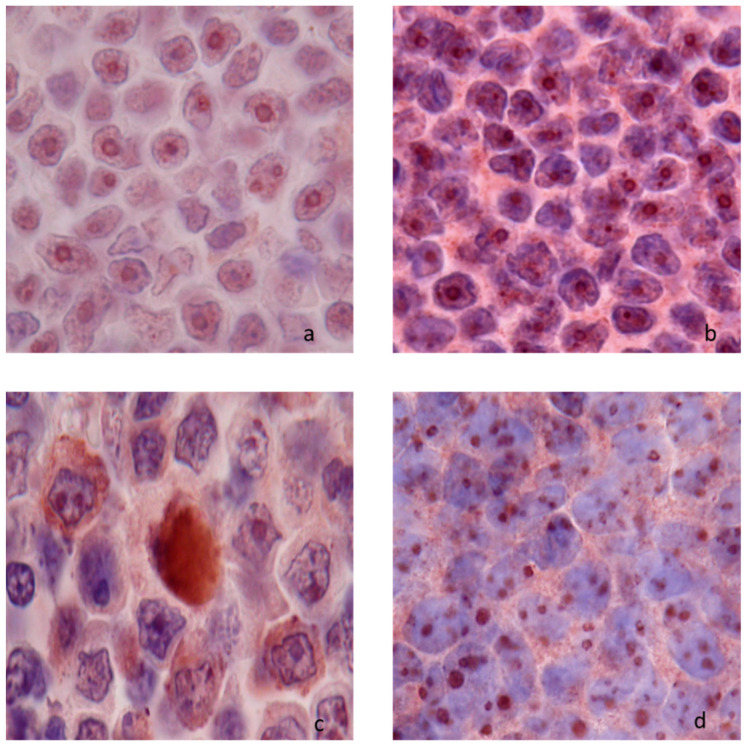
Netrin-1 immunostaining in canine lymphomas (×100) (**a**) nucleolar without cytoplasmic immunostaining (marginal zone B-cell lymphoma); (**b**) nucleolar and polar granular cytoplasmic (++) immunostaining (small clear T-cell lymphoma); (**c**) nucleolar and diffuse cytoplasmic (++) immunostaining (large granular T-cell lymphoma); (**d**) and nucleolar and cytoplasmic (+++) immunostaining (immunoblastic B-cell lymphoma).

One hundred and sixty-nine canine nodal lymphomas were tested. Two patterns of netrin-1 expression were identified: nucleolar and cytoplasmic patterns. The cytoplasmic pattern was polar or diffuse (Figure 2).

In B-cell nodal lymphomas a nucleolar immunostaining was present in all cases, without differences between low-grade and high-grade subtypes. In low-grade B-cell nodal lymphomas, cytoplasmic expression of netrin-1 was observed in a minority of cases (23.1%). In high-grade B-cell nodal lymphomas, netrin-1 immunostaining was present in the majority of cases (50.6%). This difference of netrin-1 cytoplasmic expression between low- and high-grade B-cell lymphomas was significant (*p*< 0.001) (Table 3 and Table 4).

In T-cell nodal lymphomas, netrin-1 nucleolar expression was present in 64.3% of low-grade T-cell lymphomas and in 56.2% of high-grade T-cell lymphomas; this difference of netrin-1 nucleolar expression between low- and high-grade T-cell lymphomas was not significant (*p* = 0.91).

An increase in cytoplasmic expression of netrin-1 in high-grade (78.8% of cases were positive) compared with low-grade (50.0% of cases were positive) T-cell lymphomas was present although not significant (*p* = 0.052) (Table 5 and Table 6).

### 3.2. Netrin-1 Quantification in Normal Lymph Node and in Nodal Lymphoma

#### 3.2.1. Flow Cytometry Analysis

**Figure 3:**
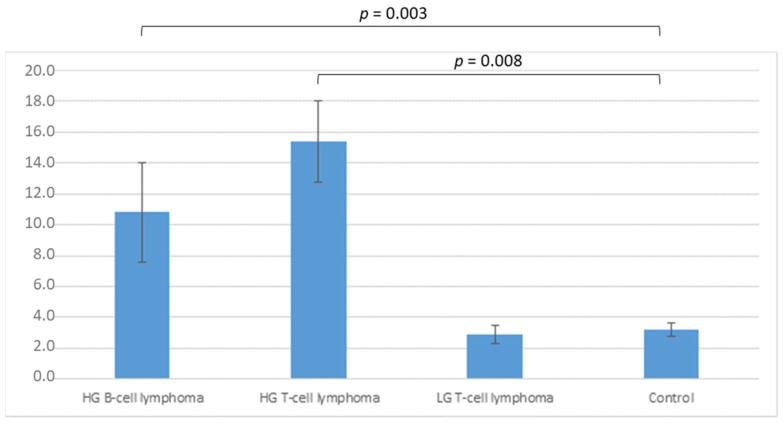
Gain in intracellular netrin-1 expression in nodal lymphomas by flow cytometry. (HG: high-grade; LG: low-grade).

Twenty six cases of nodal lymphomas and five normal lymph nodes were included and tested via flow cytometry (cf. Table S1). Netrin-1 was expressed in all cases, both lymphomas and controls. Differences were observed in fluorescence intensity. Evaluation of ΔMFI is shown in Figure 3. Differences were significant when comparing either high-grade B-cell lymphomas (mean 10.8 a.u.) or high-grade T-cell lymphomas (mean 15.4 a.u.), with control samples (mean 3.2 a.u.) (*p* = 0.003 and *p* = 0.008, respectively). No significant difference was observed between control and low-grade T-cell lymphomas (mean 2.9 a.u.) (Figure 3).

Both in B-cell type and T-cell type, netrin-1 expression was increased in high-grade lymphomas.

#### 3.2.2. RT-qPCR Analysis

**Figure 4:**
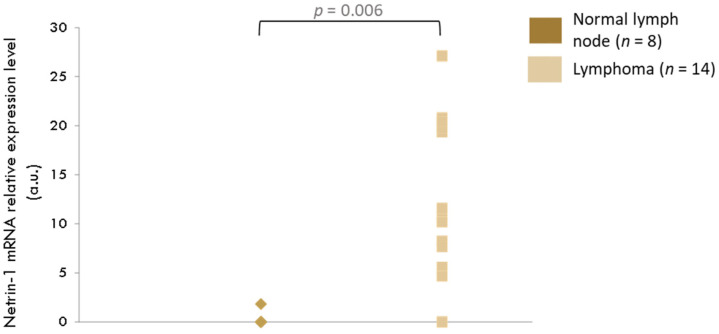
Gain of netrin-1 expression level in lymph nodes tumoral samples.

Fourteen samples of nodal lymphoma and eight samples of lymph nodes from healthy dogs were analyzed by RT-qPCR. Results are shown in Figure 4. A significant increase in netrin-1 expression level in lymph nodes with lymphoma compared with control organs was present (*p* = 0.0006)

### 3.3. In Vitro effect of Netrin-1 on Lymphoma Cells

#### 3.3.1. Effect on Lymphoma Cell Line

**Figure 5:**
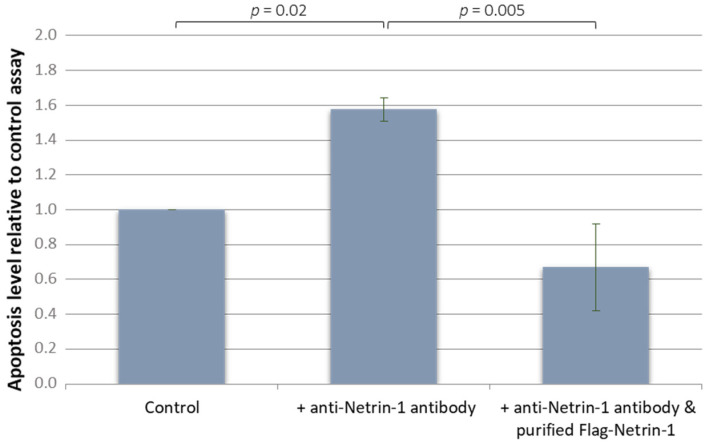
Effect of addition of anti-netrin-1 on a T-lymphoma canine cell line (PER-VAS).

On PER-VAS cell line, we measured caspase 3 activity in treated and untreated cells in serum free medium. Addition of netrin-1 interfering monoclonal antibody led to a significant increase in caspase-3 activity at 24 h compared with control condition (*p* = 0.02) (Figure 5). Addition of purified FLAG-tagged netrin-1 in the culture medium restored caspase-3 activity to control levels, with a significant decrease in apoptosis level compared with the netrin-1 interfering monoclonal antibody alone (*p* = 0.05). Thus netrin-1 seems to act as a survival factor for these neoplastic cells in vitro.

#### 3.3.2. Effect on Primary Canine Nodal Lymphoma Cell

**Figure 6:**
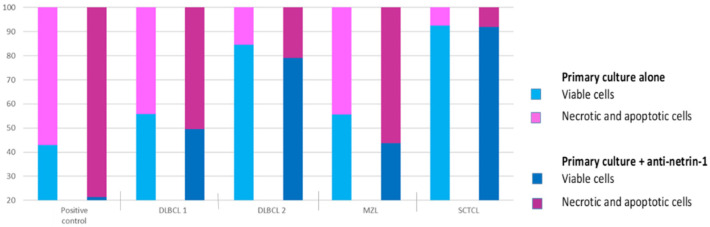
Effect of a netrin-1 interfering monoclonal antibody, in primary culture cells from nodal lymphomas. Results are presented as percentage of viable and necrotic and apoptotic cells (DLBCL: diffuse large B-cell lymphoma; MZL: marginal zone lymphoma (B-cell); SCTCL: small clear T-cell lymphoma).

On the four primary canine nodal lymphoma cells tested, a strong activation of apoptosis was noted for the positive control assay with staurosporin, with a 50% decrease in living cells after a 24 h incubation. For the two DLBCL primary cultures, cell viability was slightly decreased with a netrin-1 interfering monoclonal antibody, blocking netrin-1 binding to its dependence receptors, compared with controls (56% and 84% compared with 49% and 79%, respectively). Cell viability was also decreased in the primary cultures from the transforming marginal zone lymphoma (56% versus 43%). No effect of the netrin-1 interfering monoclonal antibody was observed for the small clear cells T-cell lymphoma (Figure 6).

## 4. Discussion

The present study examined the expression of netrin-1 in normal canine lymphoid tissues and tissues with nodal lymphoma through immunohistochemistry, flow cytometry, and RT-qPCR. To our knowledge, it is the first time that the dependence receptors concept has been investigated in canine tumor biology.

In dogs, immunohistochemistry exhibited that normal lymphoid cells may express netrin-1 but only at a nucleolar level whereas nodal lymphomas cells may also express netrin-1 at a cytoplasmic level. In fact, if the absence of nucleolar expression was regularly observed, in similar proportions between low- and high-grades, in both B and T nodal lymphoma cells, a significant increase in cytoplasmic netrin-1 expression in high-grade, compared with low-grade, was present in B-cell nodal lymphomas (the increase in high-grade T-cell lymphomas was not significant). When quantifying the expression of netrin-1 in nodal lymphomas by flow cytometry, we also observed a significant increase in netrin-1 expression in both B-cell and T-cell high-grade lymphomas (different subtypes pooled together) in comparison with low-grade lymphomas and non-lymphomatous lymphoid cells.

Nucleolar expression was never reported in embryonic or adult human or murine cells (in intestine, lung or kidney for instance [31,32,33]), in which cytoplasmic expression alone was present. It was demonstrated that in human tumoral cell lines, two isoforms of netrin-1 can be detected. The first was a truncated one, located in the nucleolus, and a complete one, with a cytoplasmic location [34]. It was thus suggested that netrin-1 can play a part in cell proliferation. When the cell divides, the nucleolus disassembles with redistribution of proteins around condensed chromosomes [35]. This can explain immunohistochemical expression of netrin-1 in cytoplasm of dividing cells.

Some proteins, such as Cip/Kip proteins with a nuclear localization, play a part in cell cycle regulation, and can display a cytoplasmic relocation in the case of neoplastic transformation. In cells from lung and colon cancers, 40% and 35%, respectively, of neoplastic cells present this pattern of expression, which is absent in healthy tissues [36,37].

In cells from canine lymphomas, this type of post-transduction alterations in netrin-1 can be considered, redirecting this protein in the cytoplasm of cells, thus inhibiting pro-apoptotic proteins.

In humans, expression of netrin-1 detected by RT-qPCR in mantle cell lymphomas and DLBCL was increased compared with tonsillar non-neoplastic samples (14 and 4.2 times superior, respectively) [16]. DLBCL encompasses several types of lymphoma, both in humans and dogs [19]. In our study, centroblastic polymorphic and immunoblastic subtypes belong to this category with some displaying cytoplasmic expression of netrin-1, paralleling was what observed in humans. Surprisingly, the only canine mantle cell lymphoma available in our immunohistochemical study displayed no expression (neither nucleolar nor cytoplasmic). No data have been published about humans regarding expression of netrin-1 in T-cell lymphomas. In canine T-cell lymphomas, an increased netrin-1 cytoplasmic expression in high-grade subtypes was present, especially in aggressive large granular T-cell lymphomas, a subtype with poor prognosis. Netrin-1 expression can thus be considered as a marker of aggressiveness in canine lymphomas. In humans, association between increased expression and high-grade tumors was noted in several other tumor types, including: 47% in lung cancer, 60% in metastatic breast cancers, 38% in neuroblastomas, ovarian, and pancreatic cancers [38,39,40,41,42].

This gain of netrin activity was proven to be a selective advantage for tumor cells survival, as downregulation of netrin-1 expression with siRNAs or interference in netrin-1 binding to its receptors via use of decoy recombinant peptides are associated with tumor cell death, both in vitro and in vivo [16,38,39,40,43]. More recently a blocking anti-netrin-1 mAb was developed and is currently being tested in phase 2 clinical trials in patients with advanced solid cancer.

Interestingly, a cell death assay realized on one canine cell line available showed that by blocking netrin-1 binding to its receptors, it was possible to induce apoptosis of tumoral cells. This cell-line is derived from a spontaneously occurring aggressive T-cell lymphoma with large granular cell morphology [24]. The aggressiveness of this T-cell lymphoma is responsible for a poor efficiency of conventional treatments. Therefore, blocking netrin-1 seems a promising way in the therapeutic perspectives of such lymphomas.

Moreover, in three of the four primary cultures available (two DLBCL and one MZL), viability was decreased in a medium with the netrin-1 interfering monoclonal antibody. The only primary T-cell lymphoma available did not show a difference. However, as these experiments were only made once, no conclusion can be drawn.

This study focuses on netrin-1, the ligand of dependence receptors, such as DCC and receptors of the UNC5 family [44]. In light of this model, imbalance in the netrin-1/receptor can be one of the underlying causes of tumor occurrence or escape, either by gain of netrin-1 expression, or by loss of receptors expression. Loss of DCC expression was detected in several cancers (stomach, prostate, ovary and testes, esophagus, breast, and hematologic malignancies) [13,44]. In human lymphoma, DCC mRNA expression was decreased in GC-DLBCL compared with control tonsils and in mantle cell lymphomas [16]. Moreover, a decreased expression of UNC5H was observed in colorectal cancers, ovarian tumors, renal tumors, lung tumors, or breast cancers [5,45]. To date, these receptors have not been investigated in dog tumors.

## 5. Conclusions

In conclusion, showed that netrin-1 was expressed in canine lymphoid cells at a nucleolar level. Cytoplasmic expression was present and increased in high-grade lymphomas compared with low-grade lymphomas. We showed that netrin-1 can act as a survival factor for neoplastic cells. Further studies are needed to better understand the possible existence of netrin-1 isoforms, to analyze netrin-1 involvement in organs other than lymph node, to evaluate the role and importance of netrin-1′s receptors, and to appreciate netrin-1 as a potential prognostic marker.

## Figures and Tables

**Table 1:** Correspondence between percentage of immunostained neoplastic cells and semi-quantitative level of expression.

Semi-Quantitative Level of Expression	Percentage of Immunostained Neoplastic Cells
0	No expression
+	<5%
++	5–50%
+++	>50%

**Table 2:** Primers sequences of canine ortholog of netrin-1.

	Primer Sequence (Forward) (5′→3′)	Tm (°C)	Primer Sequence (Reverse) (5′→3′)	Tm (°C)
Netrin-1	CGAGTGCGTGGGTGATGTAACTGC (24)	66.1	GTGGCAATCGCAGGCTTTGCAGGCC (25)	69.5
Hprt [27]	AGCTTGCTGGTGAAAAGGAC (20)	56.0	TTATAGTCAAGGGCATATCC (20)	56.0

Tm: Melting temperature.

**Table 3:** Immunohistochemical detection of netrin-1 in canine B-cell lymphomas.

Grade	Morphological Type	*n*	Nucleolar Expression		Cytoplasmic Expression			
			0	+	0	+	++	+++
Low-grade	Lymphoplasmacytic	4	0	4	3	0	1	0
	Small cell	4	0	4	1	1	2	0
	Follicular	3	1	2	2	0	1	0
	Mantle cell	1	1		1	0	0	0
	Marginal zone	14	2	12	13	0	1	0
High-grade	Centroblastic monomorphic	1	0	1	1	0	0	0
	Centroblastic polymorphic	52	5	47	23	16	13	0
	Immunoblastic	16	2	14	12	1	2	1
	Burkitt-like	8	2	6	2	1	3	2
	Small cells NOS	6	3	3	3	1	2	0

(*n* = number of samples)

**Table 4:** Immunohistochemical detection of netrin-1 in canine low-grade and high-grade B-cell lymphomas.

Grade	*n* (%)	Nucleolar Expression		Cytoplasmic Expression	
		0	+	0	Presence
Low-grade	26 (100)	4 (15.4)	22 (84.6)	20 (76.9)	6 (23.1)
High-grade	83 (100)	12 (14.4)	71 (85.6)	41 (49.4)	42 (50.6)

B-cell lymphomas were grouped in two categories: low-grade and high-grade, in absolute and relative (%) numbers. All cases with cytoplasmic expression were grouped together (*n*= number of samples).

**Table 5:** Immunohistochemical detection of netrin-1 in canine T-cell lymphomas.

Grade	Morphological Type	*n*	Nucleolar Expression		Cytoplasmic Expression			
			0	+	0	+	++	+++
	Lymphoblastic	13 *	4	8	5	2	6	0
Low-grade	Pleomorphic small cell	1	1	0	1	0	0	0
	Small clear cell (T-zone)	13	4	9	6	4	3	0
High-grade	Pleomorphic mixed	6	1	5	0	4	2	0
	Aggressive large granular cell	20 *	9	10	3	3	12	2
	Plasmacytoid	2	2		1	1	0	0
	Anaplastic	3	1	2	1	0	2	0
	Immunoblastic	2	1	1	2	0	0	0

(*n* = number of samples) * One with no visible nucleoli.

**Table 6:** Immunohistochemical detection of netrin-1 in canine low-grade and high-grade T-cell lymphomas.

Grade	*n* (%)	Nucleolar Expression		Cytoplasmic Expression	
		0	+	0	Presence
Lymphoblastic	13 * (100)	4 (33.3)	8 (66.7)	5 (38.4)	8 (61.6)
Low-grade	14 (100)	5 (35.7)	9 (64.3)	7 (50.0)	7 (50.0)
High-grade	33 * (100)	14 (43.8)	18 (56.2)	7 (21.2)	26 (78.8)

T-cell lymphomas were grouped in two categories: low-grade and high-grade, in absolute and relative (%) numbers. All cases with cytoplasmic expression were grouped together (*n*= number of samples). * One with no visible nucleoli.

## Data Availability

The data presented in this study are available on request from the corresponding author.

## Supplementary Materials

The following supporting information can be downloaded at: https://www.mdpi.com/article/10.3390/vetsci9090494/s1, Figure S1: Netrin-1 and its dependence receptors expression (Q-RT-PCR) in a canine-T-cell line (PER-VAS) Netrin-1 and its dependence receptors expression levels were analyzed by RT-PCR. Results of PCR products migration on a 2% agarose gel is presented; Figure S2: Structure of netrin-1 (localization of the 5 different amino acids between the human and the canine orthologs); Figure S3: Netrin-1 immunostaining in canine normal lymph nodes at low magnification, showing immunostaining enforcement in subcapsular sinusal region; Table S1: Flux cytometry data of control samples and samples from dog lymphomas.
