# Supplementary material for: Implication of Netrin-1 Gain of Expression in Canine Nodal Lymphoma
Source: Vet Sci. 2022 Sep 10;9(9):494. doi: 10.3390/vetsci9090494 (PMC9501284; doi:10.3390/vetsci9090494)
Supplement: Supplementary file 1 [file vetsci-09-00494-s001.zip › vetsci-1802911-supplementary.pdf]

veterinary sciences

MDPI

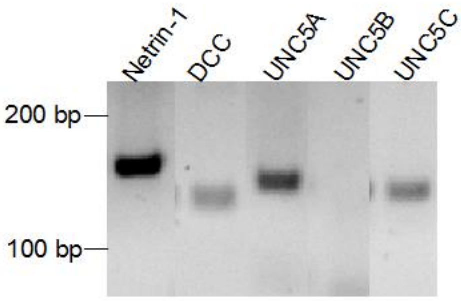

Figure S1. NETRIN-1 AND ITS DEPENDENCE RECEPTORS EXPRESSION (Q-RT-PCR) IN A CANINE-T-CELL LINE (PER-VAS) Netrin-1 and its dependence receptors expression levels were analysed by RT-PCR. Results of PCR products migration on a 2% agarose gel is presented.

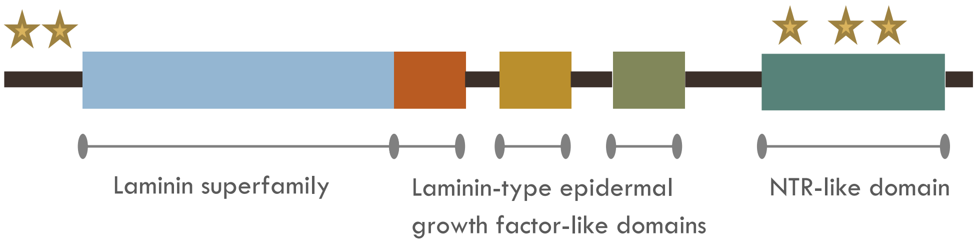

Figure S2. STRUCTURE OF NETRIN-1.(localization of the 5 different amino acids between the human and the canine orthologs.).

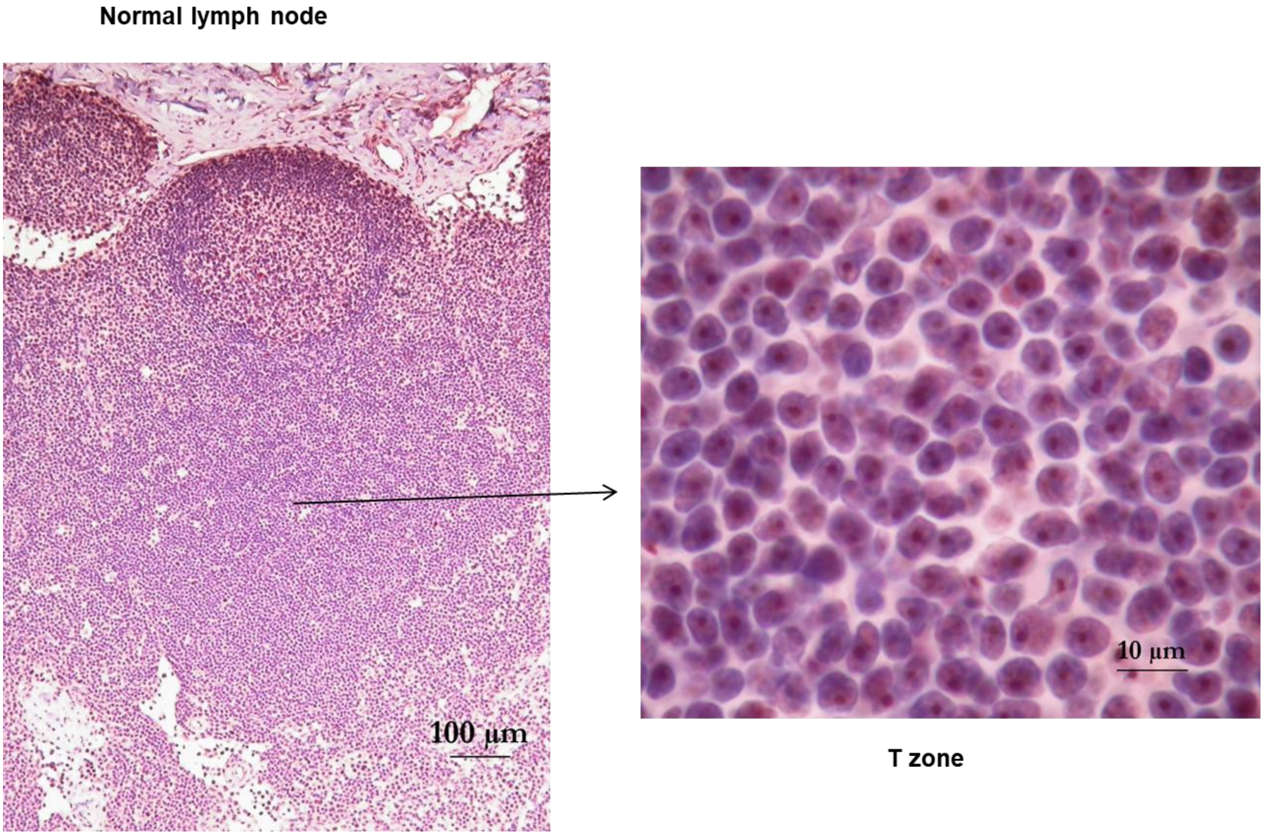

Figure S3. Netrin-1 immunostaining in canine normal lymph nodes at low magnification, showing immunostaining enforcement in subcapsular sinusal region. A zoom on cortical T-zone is present showing nucleolar immunostaining.

Table S1. Fux cytometry data of control samples and samples from dog lymphomas.

Flux cytometry							
	**Sampling**	**Diagnosis**	**Grade (L : Low / H : High)**	**% of Positive Cells**	**MFI**		
					**Control**	**Netrin-1**	**Δ MFI**
**B-cell lymphomas = 19 cases**							
TILLY	19/06/2014	small cell NOS	H	99,2	6706	159736	22,8
LASSEIGNE	03/11/2014	Immunoblastic	H	99,4	8308	197192	22,7
DEROLEZ	05/01/2015	Immunoblastic	H	92,7	5775	37540	5,5
GUIGARD	26/01/2015	Immunoblastic	H	98,9	3615	38538	9,7
MARKIEWITZ	07/01/2015	Immunoblastic	H	98,8	4350	33020	6,6
PAIR	03/02/2015	Immunoblastic	H	96,7	6113	40572	5,6
DELORME	09/04/2015	Transforming Marginal Zone	H	93	4546	18101	3,0
DANTHONY	06/04/2015	Polymorphic centroblastic	H	70	5464	29759	4,4
MALATY	16/09/2015	Immunoblastic	H	91,5	7554	48309	5,4
VIGUIER	09/06/2015	Burkitt like	H	85	4136	29260	6,1
NIOGRET	13/10/2015	Transforming Marginal Zone	H	98,5	3493	16518	3,7

SOUVIGNET	09/11/2015	Polymorphic centroblastic	H	90,8	2212	15612	6,1
GRIMOUX	09/06/2016	Polymorphic centroblastic	H	98,6	4950	103447	19,9
PIETROPAOLI	17/03/2016	Polymorphic centroblastic	H	98,7	4984	96151	18,3
INDELICATO	29/04/2016	Burkitt like	H	98,3	1663	30065	17,1
FAGET	05/12/2016	Burkitt like	H	89,1	9819	94125	8,6
BAPTISTAL	14/12/2016	Immunoblastic	H	97,5	3232	47860	13,8
CORNET	18/01/2017	Polymorphic centroblastic	H	98,9	3157	45210	13,3
MORETON	12/01/2017	Polymorphic centroblastic	H	97,9	6571	92769	13,1
**T-cell lymphomas = 7 cases**							
MOSCICKI	21/11/2014	Lymphoblastic	H	99,9	3231	51988	15,1
GOUMOT	27/11/2014	Lymphoblastic	H	99,8	5646	97681	16,3
BRIVADIS	16/02/2015	Pleomorphic, large cells	H	88,1	2715	29129	9,7
SOTTON	23/02/2016	Immunoblastic	H	98,3	3427	86707	24,3
BAUTHIER	18/05/2016	non classified	H	98,5	2764	42445	14,4
ROLLAND	01/10/2015	subcutaneous	B	85,5	3980	11278	1,8
JACQUIER	29/11/2016	mucous	B	92,4	16777	82702	3,9
**Control lymph node = 5 cases**							
Beagle1 MdG	26/10/2015			90,5	3034	11904	2,9
Beagle1 MdD	26/10/2015			85	3011	12561	3,2
Beagle2 MdG	26/10/2015			90,2	2851	14335	4,0
Beagle2 MdD	26/10/2015			84	2764	7321	1,6
SOLIVERES	12/02/2015			94,2	2688	14367	4,3
